# Characterization and Control of *Dendrobium officinale* Bud Blight Disease

**DOI:** 10.3390/pathogens12040621

**Published:** 2023-04-20

**Authors:** Jinzhao Zhang, Haodong Sha, Weiliang Chen, Bizeng Mao

**Affiliations:** 1 Institute of Biotechnology, Zhejiang University, Hangzhou 310058, China; 2 Ministry of Agriculture Key Lab of Molecular Biology of Crop Pathogens and Insects, Hangzhou 310058, China; 3 Key Laboratory of Biology of Crop Pathogens and Insects of Zhejiang Province, Hangzhou 310058, China

**Keywords:** bud blight, *Dendrobium officinale*, MLSA, Meitian

## Abstract

*Dendrobium officinale* is an important traditional Chinese medicine (TCM). A disease causing bud blight in *D. officinale* appeared in 2021 in Yueqing city, Zhejiang Province, China. In this paper, 127 isolates were obtained from 61 plants. The isolates were grouped into 13 groups based on collected areas and morphological observations. Four loci (ITS, LSU, *tub2* and *rpb2*) of 13 representative isolates were sequenced and the isolates were identified by constructing phylogenetic trees with the multi-locus sequence analysis (MLSA) method. We found the disease to be associated with three strains: *Ectophoma multirostrata*, *Alternaria arborescens* and *Stagonosporopsis pogostemonis*, with isolates frequencies of 71.6%, 21.3% and 7.1%, respectively. All three strains are pathogenic to *D. officinale*. *A. arborescens* and *S. pogostemonis* isolated from *D. officinale* were reported for the first time. Iprodione (50%), 33.5% oxine-copper and Meitian (containing 75 g/L pydiflumetofen and 125 g/L difenoconazole) were chosen to control the dominant pathogen *E. multirostrata*, with an EC_50_ value of 2.10, 1.78 and 0.09 mg/L, respectively. All three fungicides exhibited an effective inhibition of activities to the growth of the dominant pathogen *E. multirostrata* on potato dextrose agar (PDA) plates, with Meitian showing the strongest inhibitory effect. We further found that Meitian can effectively control *D. officinale* bud blight disease in pot trial.

## 1. Introduction

*Dendrobium officinale* Kimura et Migo is a famous traditional Chinese medicine, which contains many bioactive components, such as polysaccharides, alkaloids, flavonoids and phenanthrene phenols [1,2]. *D. officinale* has been found to have functions related to immunity enhancement, the lowering of blood sugar, blood lipids and blood pressure, anti-oxidation, anti-tumor and cancer cell inhibition [3].

In nature, *D. officinale* grows in shady and humid rock crevices, and grows symbiotically with lichens, mosses, ferns and other plants [1]. It has been cropped in several provinces in China, such as Zhejiang, Guizhou and the Yunnan Province [4]. *D. officinale* is mostly cropped in greenhouses using pine scales, sawdust, wood and rocks as media, with drip and spray irrigation systems supplying water and fertilizer. Greenhouses maintain suitable temperature and humidity levels for the growth of *D. officinale*, as well as pathogens.

Several *D. officinale* diseases are caused by fungal pathogens, some of them occurring on leaves, such as black spot disease caused by *Alternaria arborescens*, *A. alternata* and *Cladosporium oxysporum* [5,6,7]; anthracnose disease caused by *Colletotrichum gloeosporioides* [8]; or botrytis disease caused by *Botrytis cinerea* [9]. Leaf spots can be caused by *Phoma multirostrata* var. *microspora*, *Neopestalotiopsis clavispora* and *Cladosporium cladosporioides* [10,11,12].

Stem diseases, such as stem dieback, can be caused by *A. alternata* [13] and *Fusarium spp.* [14,15], and stem rot can be caused by *Lasiodiplodia theobromae*, *F. kyushuense* and *Sclerotium rolfsii* [16,17,18]. Root rot disease can be caused by *F. sambucium* and *F. chlamydosporum* [19]. *F. oxysporum* causes *Fusarium* wilt disease [20]. Ring rot disease caused by *Myrothecium roridum* has appeared on leaves and stems [21], and soft rot disease caused by *F. oxysporum* and *Epicoccum* can afflict stems and roots [22,23].

*D. officinale* yield is seriously threatened by the bud blight disease, which has been a severe problem in many plantation areas since 2021, especially in Yueqing City, Zhejiang Province, China. The disease incidence was over 50% in some greenhouses. Unfortunately, there are currently no effective countermeasures available. Little is known about the species causing the bud blight disease in *D. officinale* in China or in other parts of the world. Normally, integrated disease management strategies are employed to reduce the incidence rate of the disease. Chemical fungicide application is one such method that is common and effective in controlling disease [24].

Three commonly used fungicides with different inhibition mechanisms, iprodione, oxine-copper and Meitian, have been chosen to control isolates in this study. Iprodione inhibits protein kinases and controls intracellular signaling for many cellular functions, resulting in the inhibition of fungal growth [25]. Oxine-copper releases copper ions to combine with the protein of fungi, causing the protease denaturation and function loss, therefore effectively inhibiting hyphal development [26]. Pydiflumetofen, newly created by Syngenta, is a succinate dehydrogenase inhibitor (SDHI). SDHI fungicides bind to the succinate dehydrogenase (SDH) complex and block the transport of electrons in the respiratory chain which are necessary to produce ATP, curtailing energy production and arresting fungal growth [27]. Difenoconazole is a sterol demethylation inhibitor that inhibits cell membrane ergosterol biosynthesis [28].

Little is known about *D. officinale* bud blight disease, the morphological and biological characteristics of the pathogens, or the control method. This study aims to identify and characterize the disease and its associated pathogens, and find a way to control *D. officinale* bud blight disease.

## 2. Materials and Methods

### 2.1. Plant Materials, Pathogens Isolation and Purification

From 2021 to 2022, *D. officinale* plants (*n* = 61) with bud blight disease were collected from a greenhouse in Yueqing City (28.07° N, 120.57° E), Zhejiang Province, China. The incidence rate of the disease was assessed by visual observation of the presence or absence of symptomatic plants in the surveyed greenhouses. Pathogens were isolated according to the following method: the symptomatic plants were cut with a sterilized scalpel and rinsed with tap water for 15 min to remove dirt from the surface, then dried on tissue paper. Afterward, the symptomatic buds were cut into 4 mm^2^ segments using a sterilized scalpel, superficially disinfected with 5% sodium hypochlorite solution (0.25% active ingredient of chlorine) for 1 min and 75% alcohol for 30 s, then washed with sterile distilled water 3 times, dried on sterile filter papers under aseptic conditions, and finally, the picked segments were placed onto PDA plates. The plates were subsequently incubated at 25 °C; in the dark, and the colonies were purified by the hyphal tip method [29] and then subcultured on the PDA and oatmeal agar (OA) media for morphological observation.

### 2.2. Pathogenicity Tests of Isolates

To test for pathogenicity, the fungal isolates were inoculated on the original host. The top three leaves were inoculated. The leaves were stabbed gently with sterile needles to cause tiny wounds, and the mycelial plugs (∅ = 6 mm) from 5-day-old cultures of the isolates were placed on the surfaces of the wounded leaves and wrapped with cling wrap. In contrast, the control plants received non-colonized agar plugs. All plants were covered with plastic bags to maintain moisture and then placed in a light incubator under conditions of 25 °C, 12 h dark/light. Each treatment had 3 replicates. All inoculated plants were observed for 20 days. Isolates causing necrosis over 4 mm^2^ were considered pathogenetic. Fungal isolates which were re-isolated from inoculated plants were identified by *rpb2* sequence data to fulfill Koch’s postulates.

### 2.3. Identification of Pathogens

#### 2.3.1. Morphological Observation

Purified isolates were grown on PDA and OA media at 25 °C in the dark for 7 days, after which morphological characteristics were observed and photographed. The microstructures of isolates were observed with a Nikon Eclipse Ni microscope with differential interference contrast (DIC) optics, equipped with a Nikon DS-Fi2 digital camera [30] and a jsz6 dissecting microscope. If necessary, near-UV light was used to promote the production of conidia [31].

#### 2.3.2. DNA Extraction, Amplification and Sequencing

The genomic DNA was extracted using the method described by Pan Li et al. [32].

According to the manufacture’s instruction, the following four loci were amplified using a 2×Phanta Flash Master Mix kit (Vazyme, Nanjing, China): internal-transcribed spacer (ITS), ribosome large subunit rRNA gene (LSU), beta-tubulin (*tub2*) and RNA polymerase II second largest subunit (*rpb2*) [33]. Primers for the four loci were ITS5/ITS4 [34,35] for ITS, LROR/LR5 [36,37] for LSU, BT2A/BT2B [38] for *tub2* and RPB2-5F2/fRPB2-7cR [39,40] for *rpb2* (Appendix A).

PCR amplifications were performed in a total volume of 25 μL containing 13 μL 2×PCR buffer (Vazyme, Nanjing, China), 1 μL of each primer, and 1–10 ng genomic DNA. For LSU, ITS and *tub2*, the PCR amplification condition were: an initial denaturation for 3 min at 95 °C, followed by 35 cycles of 15 s at 95 °C, 15 s at 53 °C (for LSU and ITS) or 56 °C (for *tub2*), 1 min at 72 °C, with a final extension step for 5 min at 72 °C [41]. For *rpb2*, the PCR amplification condition were: an initial denaturation at 95 °C for 3 min, followed by 5 cycles of 15 s at 95 °C, 15 s at 60 °C and 1 min at 72 °C, then 5 cycles with a 58 °C annealing temperature and 30 cycles with a 54 °C annealing temperature, and a final extension step for 5 min at 72 °C [42]. PCR products were observed on 1% agarose gel. Sanger sequencing was conducted by Youkang Biotechnology Co., Ltd. (Hangzhou, Zhejiang Province, China). The accession numbers of all generated sequences in this study were further obtained from GenBank and listed in Table 1.

#### 2.3.3. Phylogenetic Analysis

Phylogenetic constructions were made by maximum likelihood. All obtained sequences were compared in the Basic Local Alignment Search Tool (BLAST). Sequences of related species were downloaded from NCBI and listed in Tables S1–S3. Subsequent alignments for four individual loci (ITS, LSU, *rpb2* and *tub2*) were generated with MAFFT v. 7 (https://mafft.cbrc.jp/alignment/server/, accessed on 10 April 2023) using default settings on a web server [43]. Gaps were considered to be missing data and alignments were manually adjusted for maximum alignment and sequence similarity. Sequences were cut to the same length using BioEdit v. 7.2.5. Concatenation and maximum likelihood analyses, including 1000 bootstrap replicates, were conducted using RAxML GUI v. 2.0.6. A general time-reversible (GTR) model was applied with a gamma-distributed rate variation. The resulting trees were viewed using MEGA 11 [33].

### 2.4. Fungicides Testing for the Control of the Disease Caused by E. multirostrata

#### 2.4.1. Sensitivity Assessment In Vitro

The mycelial growth rate method [44] was used to assess the sensitivity of the pathogen to the following fungicides: 50% iprodione (FMC, Los Gatos, CA, USA), 33.5% oxine-copper (Hong Yang Chemical Industry, Lvliang, China), 200 g/L Meitian (containing 75 g/L pydiflumetofen and 125 g/L difenoconazole, Syngenta, Nantong, China). 

Fungicides were added to the PDA plate at final concentrations of 156.25, 31.25, 6.25, 1.25 and 0.25 mg/L for 50% iprodione; 33.5, 6.7, 1.34, 0.268 and 0.0536 mg/L for 33.5% oxine-copper; and 2, 0.4, 0.08, 0.016 and 0.0032 mg/L for Meitian (containing 75 g/L pydiflumetofen and 125 g/L difenoconazole). Mycelial plugs of the pathogen were placed at the center of the fungicide-amended PDA plates and incubated in the dark at 25 ℃ for 7 d. Plugs placed on water-amended PDA plates served as the control. Each treatment had three replicates. The colony diameter was measured to evaluate the sensitivity of the pathogen to fungicide. Variance analysis and calculation of EC_50_ values were performed using IBM SPSS Statistics v. 26 [44].

#### 2.4.2. Control Test In Vivo

Healthy *D. officinale* plants were inoculated with pathogens using the same method in the pathogenicity tests of the isolates described above. When infective symptoms initially appeared, plants were removed from sampling bags for hours to dry. Of the Meitian (recommended minimum concentration in the field), 80 mg/L was evenly sprayed on the surface of the plants, and then whole pots of plants were put back in the incubator with a sampling bag to retain moisture. The control treatment was sprayed with an equal volume of sterile water. Each treatment had 6 replicates. The observation was carried out 20 days after inoculation.

## 3. Results

### 3.1. Field Observation of Disease

**Figure 1:**
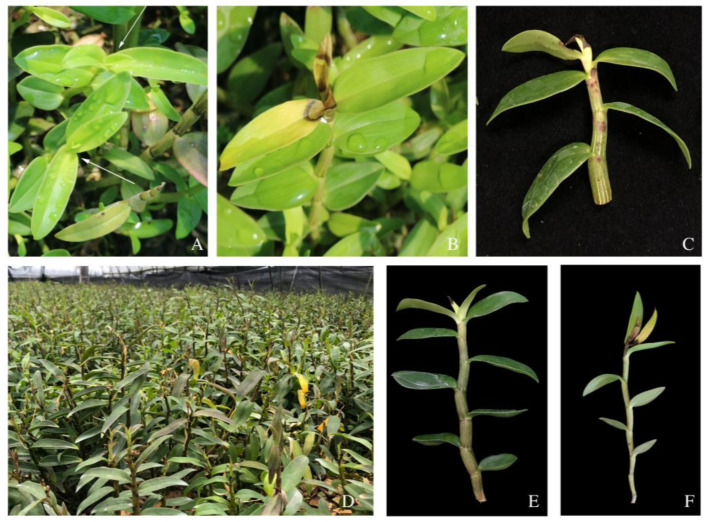
The symptoms of *Dendrobium officinale* bud blight: (**A**) healthy plants (white arrows pointing); (**B**–**F**) diseased plants.

In September 2021, *D. officinale* bud blight disease was found in Yueqing City, Zhejiang Province, China. It causes young buds to turn yellow and develop blight lesions which can spread to new leaves. Ultimately, the buds and 3 to 5 top leaves wither and the plants stop growing (Figure 1). As far as we know, this study is reporting the disease, which we named *Dendrobium officinale* “bud blight” according to the symptoms, for the first time.

The disease mostly occurs from June to July, and September to October. During these periods, high temperatures, high humidity, and poor ventilation are conducive to the growth and reproduction of pathogens. The disease spreads rapidly in some greenhouses and the disease incidence was calculated to be over 50% using a random sample of 100 plants.

### 3.2. Grouping of Isolates and Phylogenetic Analysis

A total of 127 fungal isolates were isolated from 61 diseased plants and based on isolates’ collected area and morphological traits, were grouped into 13 groups. Thirteen representative isolates were selected for further analysis. Each isolate came from different infected buds or leaves. Four loci (ITS, LSU, *rpb2* and *tub2*) of the 13 representative isolates were sequenced and the accession numbers were listed in Table 1. Consistent with their morphological traits and ITS sequences, these fungi belong to three genera, encompassing *Ectophoma*, *Alternaria* and *Stagonosporopsis*, with frequencies of 71.6%, 21.3% and 7.1% (Table 2), respectively.

**Figure 2:**
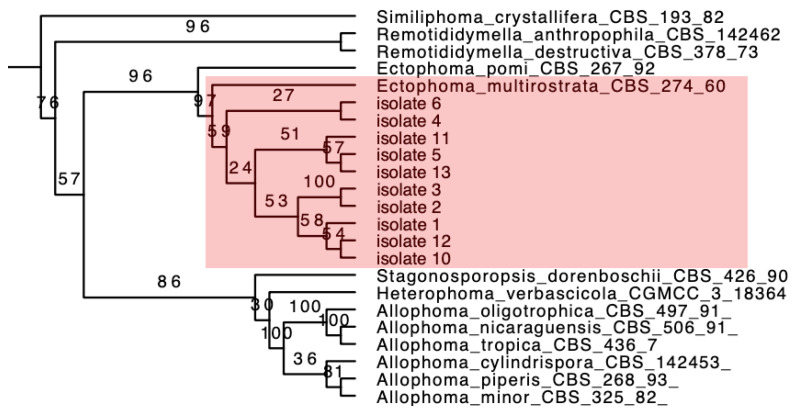
Partial phylogenetic tree inferred from the maximum likelihood analysis, based on a concatenated alignment of ITS, LSU, *tub2* and *rpb2* sequences to identify the *Ectophoma* strains. Ten isolates and *Ectophoma multirostrata* are in one clade (shades of red). The ML bootstrap support is posted on the branches.

Further, maximum likelihood, phylogenetic trees were built using the MLSA method to identify pathogens at the species level. For *Ectophoma* strains, the final concatenated DNA sequence dataset comprised 150 isolates and consisted of 2205 characters, including alignment gaps (gene boundaries ITS: 649 bp, LSU: 680 bp, *rpb2*: 601 bp, *tub2*: 275 bp). *Neocucurbitaria quercina* (CBS 115095) served as an outgroup (Figure S1). According to the phylogenetic tree (Figure 2), these 10 isolates were identified as *Ectophoma multirostrata*. The topology of the phylogenetic tree is consistent with N. Valenzuela-Lopez’s research [33]. The full phylogenetic tree is in Figure S1.

For *Alternaria* strains, the final concatenated DNA sequence dataset comprised 113 isolates and consisted of 1748 characters, including alignment gaps (gene boundaries ITS: 466 bp, LSU: 851 bp, *rpb2*: 431 bp). *Cicatricea salina* (CBS 302 84) served as an outgroup (Figure S2). For *Stagonosporopsis* strains, the final concatenated DNA sequence dataset comprised 50 isolates and consisted of 2054 characters, including alignment gaps (gene boundaries ITS: 497 bp, LSU: 709 bp, *rpb2*: 596 bp, *tub2*: 252 bp). *Allophoma piperis* (CBS 268 93) served as an outgroup.

**Figure 3:**
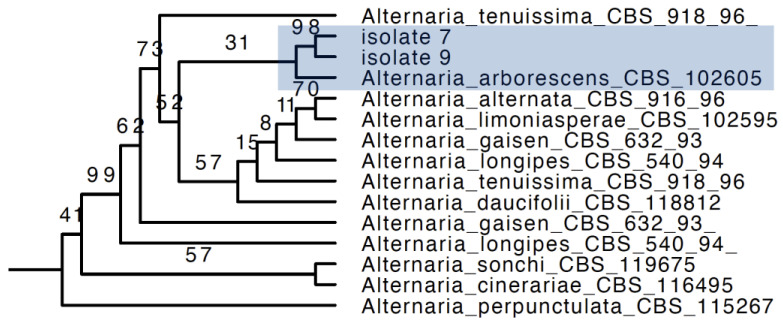
Partial phylogenetic tree inferred from the maximum likelihood analysis, based on a concatenated alignment of ITS, LSU, *tub2* and *rpb2* sequences to identify the *Alternaria* strains. Two isolates and *Alternaria arborescens* are in one clade (shades of blue). The RAxML bootstrap support is posted on the branches.

**Figure 4:**
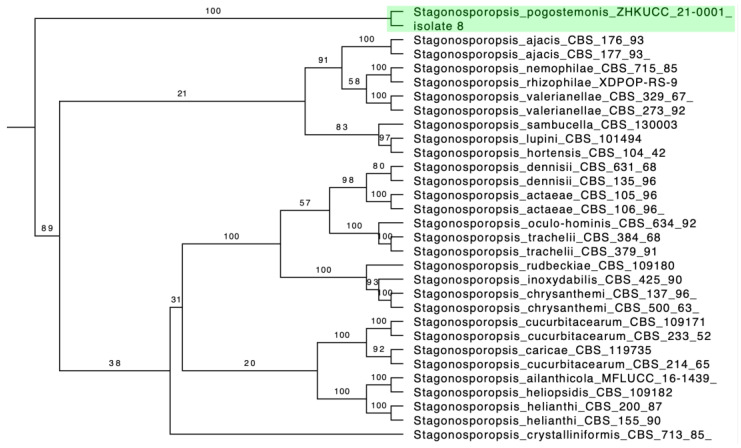
Partial phylogenetic tree inferred from the maximum likelihood analysis, based on a concatenated alignment of ITS, LSU, *tub2* and *rpb2* sequences to identify the *Stagonosporopsis* strains. One isolate and *Stagonosporopsis pogostemonis* are in one clade (shades of green). The RAxML bootstrap support is posted on the branches.

According to the phylogenetic trees, two isolates (isolate 7 and 9) were identified as *Alternaria arborescens* (Figure 3) and isolate 8 as *Stagonosporopsis pogostemonis* (Figure 4). The full phylogenetic trees of *Alternaria arborescens* and *Stagonosporopsis pogostemonis* are in Figures S2 and S3, respectively.

### 3.3. Pathogenicity Assessment

**Figure 5:**
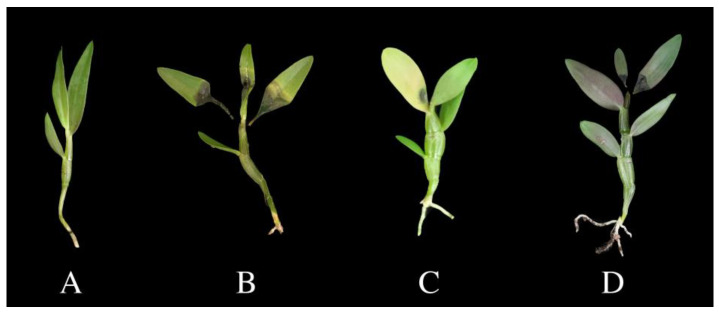
Pathogenicity test. *D.officinale* plants inoculated with (**A**) non-colonized PDA plugs; (**B**) *Ectophoma multirostrata*; (**C**) *Alternaria arborescens*; (**D**) *Stagonosporopsis pogostemonis*.

One representative isolate from each strain was selected for the pathogenicity test. Blight symptoms were observed on buds and leaves 10 days after inoculation. The symptoms were consistent with those of the disease observed in the field. The fungal isolates were re-isolated from infected leaves, which fulfilled Koch’s postulates. No symptoms appeared in the control group (Figure 5). All three strains were pathogenetic to *D. officinale.*

### 3.4. Morphological Observation of Pathogens

**Figure 6:**
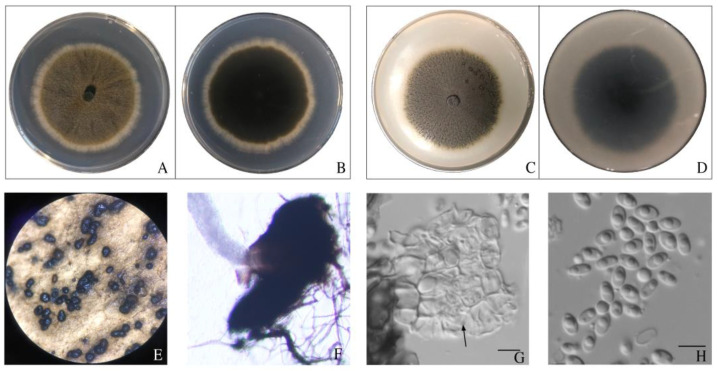
The colony of *Ectophoma multirostrata* grown on PDA, front (**A**) and back (**B**), and on OA, front (**C**) and back (**D**), for 5 days; (**E**) pycnidia grown on an OA medium; (**F**) pycnidia with conidia poured out; (**G**) conidiogenous cells (black arrow pointing); (**H**) conidia. Scale bar for (**G**,**H**) is 5 μm.

After incubation of the dominant pathogen colonies, *Ectophoma multirostrata*, on an OA medium for 7 days, we observed that the mycelia were brown; the pycnidia were globose or subglobose, brown to dark brown, solitary or confluent; the conidiogenous cells were transparent; and the conidia were transparent, nearly olive-shaped, about 1 to 1.5 × 5 to 6.5 μm in size, with one or more rounded protrusions on the surface (Figure 6).

**Figure 7:**
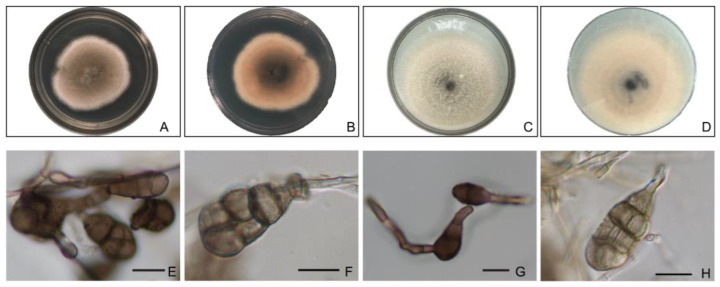
The colony of *Alternaria arborescens* grown on PDA, front (**A**) and back (**B**), and on OA, front (**C**) and back (**D**), for 5 days; (**E**–**H**) conidia. Scale bars for (**E**–**H**) are 10 μm.

*Alternaria arborescens* colonies were typically grayish to dark gray on PDA. Conidia were septate, slightly constricted near some septa, with few longitudinal septa, obclavate or ovate in shape (6.5 to 15.0 × 12.2 to 18 μm) (Figure 7).

**Figure 8:**
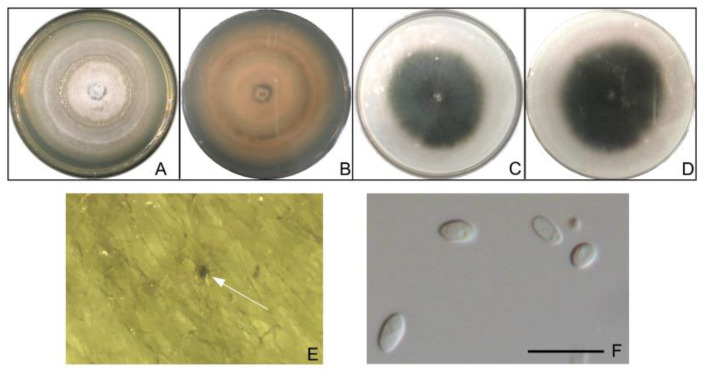
The colony of *Stagonosporopsis pogostemonis* grown on PDA, front (**A**) and back (**B**), and on OA, front (**C**) and back (**D**), for 5 days. (**E**) Pycnidia grown on an OA medium (white arrow pointing); (**F**) conidia. Scale bar for (**F**) is 10 μm.

*Stagonosporopsis pogostemonis* colonies were white on PDA, but dark gray on OA. Conidiomata were solitary and covered with dense hyphae. Conidia were 1 to 1.5 × 5 to 7 μm, olive-shaped, transparent, solitary and aseptate (Figure 8).

### 3.5. Sensitivity Assessment of Pathogens to Fungicides In Vitro

As *E. multirostrata* was the dominant pathogen in all obtained isolates, it was used as the indicator pathogen in fungicide tests.

**Figure 9:**
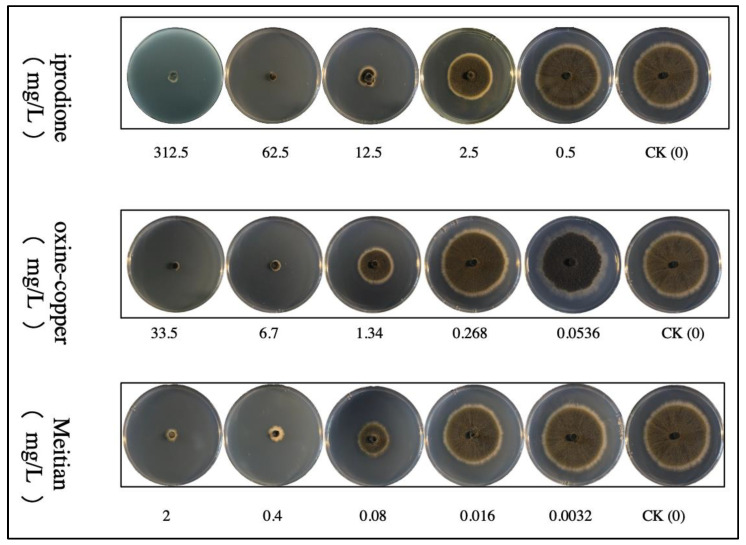
Pictures of *E. multirostrata* with different treatments. Each treatment has four replicates.

**Figure 10:**
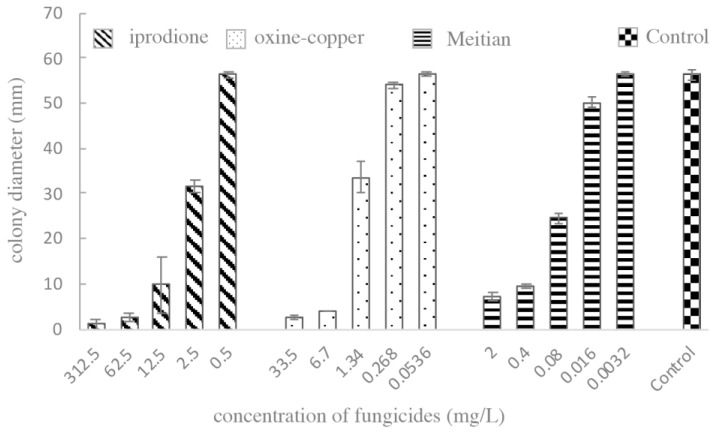
Colony diameters of *E. multirostrata* under different treatments. Each treatment has four replicates.

The diameter of mycelium gradually increased with the decrease in fungicide concentration in PDA plates (Figure 9 and Figure 10). The EC_50_ values of iprodione, oxine-copper and Meitian were 2.10, 1.78 and 0.09 mg/L, respectively. The 95% confidence intervals (CI) were 1.36–3.05, 1.43–2.24 and 0.07–0.13 mg/L, respectively (Table 3).

All three fungicides had an inhibitory effect on *E. multirostrata*, with Meitian having the strongest effect.

### 3.6. Pot Trial of Meitian against Bud Blight Disease

**Figure 11:**
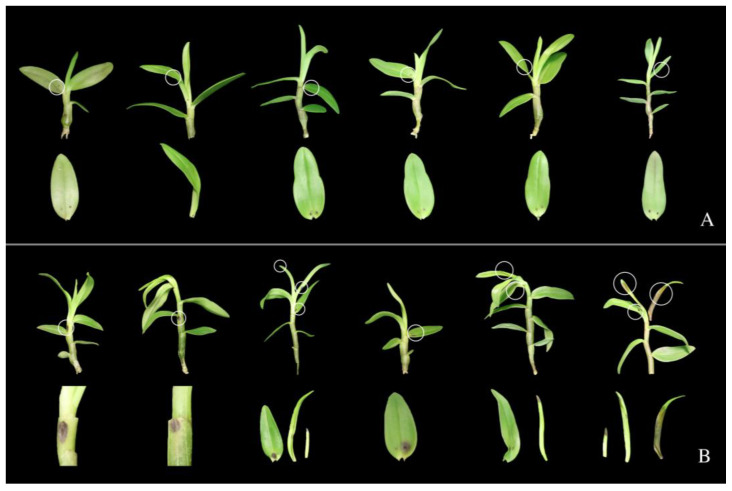
Pot trial of Meitian against bud blight disease. Plants inoculated with *E. multirostrata* were sprayed with (**A**) 80 mg/L of Meitian and (**B**) sterile water over twenty days. Inoculated leaves of plants were highlighted with white circles and exhibited below.

Meitian, as the most effective inhibitor of *E. multirostrata* among the three fungicides, was selected for the pot trial. Twenty days after inoculation with *E. multirostrata*, no symptoms were present in plants sprayed with Meitian. The disease incidence in the experimental group was 0 (Figure 11A), while blight symptoms appeared 100% in the control group (Figure 11B).

## 4. Discussion

As a traditional Chinese medicine, *D. officinale* is widely planted in multiple provinces in China and its value was over CNY 2.7 billion in 2020 [45]. However, *D. officinale* fungal diseases are becoming more serious with the increased scale of cultivation. Bud blight appeared recently with serious effects in some planting areas, but no pathogens have been reported yet. This study found that *Ectophoma multirostrata*, *Alternaria arborescens* and *Stagonosporopsis* could all cause *D. officinale* bud blight and *E. multirostrata* was the dominant pathogen.

Plant pathogen identification includes traditional and molecular methods. Traditional identification is based on morphological characteristics, growth characteristics, host range, and biochemical characteristics, etc. Molecular identification focuses on sequencing and comparison of conserved DNA sequences, such as ITS, but it is difficult to identify pathogens at the species level with individual steward genes [46]. Multi-locus sequence analysis (MLSA) is a method of aligning, cutting and joining two or more specific gene sequences to generate phylogenetic trees, and it has become a widely-accepted method in taxonomy due to its high resolution and convenience [47].

*E. multirostrata* has been reported to cause root rot disease in *Celosia argentea*, chickpea and green gram (*Vigna radiata*) [48]. *E. multirostrata* was originally classified in the genus *Phoma*, however, in an article published by N. Valenzuela-Lopez in 2018, it was classified into a new genus, *Ectophoma*, based on morphological structure and phylogeny [33]. In 2018, Xie et al. reported that *Phoma multirostrata* var. *microspora* can cause *D. officinale* leaf spot [10], with lesions appearing only on the back of the leaves and the isolate appearing white on the PDA plate. The *E. multirostrata* isolated in this study causes bud blight on *D. officinale* and appears brown on the PDA plate. Three loci which were used by Xie et al. have been sequenced: the sequence of the ITS, ACT (actin gene) and TEF (translation elongation factor) loci between the two strains share 99, 99 and 98% similarity, respectively. The difference between the two pathogens should be a topic for further research.

*A. arborescens* has been reported to cause leaf blotch and fruit spot diseases [49], as well as moldy core and core rot in apples [50,51,52]. In addition, it causes leaf spot in the purple lotus, pineapple sage, *Brassica rapa* subsp. *parachinensis*, *Symphyotrichum novi-belgii* and rice [53,54,55,56]. Additionally, *A. arborescens* causes early blight in tomato [57] and heart rot in pomegranates [58,59].

There are only a few existing reports on *S. pogostemonis*. It belongs to the genus *Phoma* and causes leaf spot and stem blight in *Pogostemon cablin* (Lamiaceae), but it has not been implicated in any disease of *D. officinale*. To the best of the author’s knowledge, this study is the first report of *S. pogostemonis* isolated from *D. officinale* causing bud blight disease.

Mirghasempour et al. reported that five *Fusarium* species can cause *D. officinale* dieback disease, with the symptoms appearing as chlorotic, blighted and wilted leaves of the apical meristem, with the shoot tip showing dark brown necrosis, dieback and eventually shoot death [14]. The dieback and bud blight could be distinguished easily from each other by symptoms: dieback disease infects from the shoot tip, while bud blight disease infects from the bud and new leaves.

Meitian is a fungicide mixed with 75 g/L pydiflumetofen and 125 g/L difenoconazole, and has the same components as Miravis Duo, which is approved in the US. Pydiflumetofen is a succinate dehydrogenase inhibitor (SDHI) that disrupts energy production [60]. Difenoconazole is a sterol demethylation inhibitor that inhibits cell membrane ergosterol biosynthesis [28]. It has been reported that Meitian can effectively inhibit rose powdery mildew and cucumber powdery mildew in the field [61,62]. In current study, Meitian was proven to be effective in controlling *D. officinale* bud blight. Due to its low-toxicity and high efficiency, Meitian is a promising tool for controlling *D. officinale* diseases.

## 5. Conclusions

Bud blight in *D. officinale* was reported for the first time in the present study. The pathogens included *E. multirostrata*, *A. arborescens* and *S. pogostemonis*. *A. arborescens* and *S. pogostemonis* were isolated from *D. officinale* for the first time. Among these pathogens, *E. multirostrata* was the dominant pathogen, with isolates accounting for 71.6% of detected pathogens. Three fungicides were tested to control *E. multirostrata* in vitro, with Meitian displaying the best inhibition effect. Further, through pot trail assessment, we found that Meitian can effectively inhibit *D. officinale* bud blight.

## Figures and Tables

**Table 1:** GenBank accession numbers of isolates obtained in this study.

Species	Strain Number	GenBank Accession Numbers			
		ITS	LSU	*tub2*	*rpb2*
*Ectophoma* sp.	1	OQ073676	OQ096504	OQ271767	OQ271782
*Ectophoma* sp.	2	OQ073677	OQ096505	OQ271768	OQ271783
*Ectophoma* sp.	3	OQ073678	OQ096506	OQ271769	OQ271784
*Ectophoma* sp.	4	OQ073679	OQ096507	OQ271770	OQ271785
*Ectophoma* sp.	5	OQ073680	OQ096508	OQ271771	OQ271786
*Ectophoma* sp.	6	OQ073681	OQ096509	OQ271772	OQ271787
*Alternaria* sp.	7	OQ073682	OQ096510	OQ271773	OQ271788
*Stagonosporopsis* sp.	8	OQ073683	OQ096511	OQ271774	OQ271789
*Alternaria* sp.	9	OQ073684	OQ096512	OQ271775	OQ271790
*Ectophoma* sp.	10	OQ073685	OQ096513	OQ271776	OQ271791
*Ectophoma* sp.	11	OQ073686	OQ096514	OQ271777	OQ271792
*Ectophoma* sp.	12	OQ073687	OQ096515	OQ271778	OQ271793
*Ectophoma* sp.	13	OQ073688	OQ096516	OQ271779	OQ271794

**Table 2:** Sampling details, number of isolates collected, and frequency of fungal species identified in the present study.

Geographic Origin	Species	Number of Isolates	Isolate Frequency (%)
Yueqing City (Zhejiang Province)	*Ectophoma* spp.	91	71.6
	*Alternaria* spp.	27	21.3
	*Stagonosporopsis* spp.	9	7.1
	Total	127	100

**Table 3:** Inhibitory effects of three fungicides on *E. multirostrata*.

*E. multirostrata*	Iprodione (mg/L)	Oxine-Copper (mg/L)	Meitian (mg/L)
EC_50_	2.10	1.78	0.09
95% CI	1.36–3.05	1.43–2.24	0.07–0.13

## Data Availability

Sequences have been deposited in GenBank (Table 1). The data presented in this study are openly available in NCBI. Publicly available datasets were analyzed in this study. These data can be found here: https://www.ncbi.nlm.nih.gov/ (accessed on 28 February 2023).

## Supplementary Materials

The following supporting information can be downloaded at: https://www.mdpi.com/article/10.3390/pathogens12040621/s1, Table S1. GenBank accession numbers of strains used to identify Ectophoma strain in the phylogenetic analyses; Table S2. GenBank accession numbers used to identify Alternaria strains used in the phylogenetic analyses; Table S3. GenBank accession numbers used to identify Stagonosporopsis strains used in the phylogenetic analyses; Figure S1. Phylogenetic tree for *Ectophoma* Strains; Figure S2. Phylogenetic tree for *Alternaria* Strains; Figure S3. Phylogenetic tree for *Stagonosporopsis* Strain.

## Appendix A

**Table A1:** Primers used in this study.

Loci	Primer	Sequence
ITS	ITS5	GGAAGTAAAAGTCGTAACAAGG
	ITS4	TCCTCCGCTTATTGATATGC
LSU	LROR	ACCCGCTGAACTTAAGC
	LR5	TCCTGAGGGAAACTTCG
*tub2*	BT2A	GGTAACCAAATCGGTGCTGCTTTC
	BT2B	ACCCTCAGTGTAGTGACCCTTGGC
*rpb2*	RPB2-5F	GAYGAYMGWGATCAYTTYGG
	RPB2-7cR	CCCATRGCTTGYTTRCCCAT

## References

[1] Zhang Y. (2016). Screening of Anthracnose Resistant Germplasm Resources of *Dendrobium officinale* and Seed Production and Application of Resistant Strains. Master’s Thesis.

[2] Luo Q., Tang Z., Zhang X., Zhong Y., Yao S., Wang L., Lin C., Luo X. (2016). Chemical Properties and Antioxidant Activity of a Water-Soluble Polysaccharide from *Dendrobium Officinale*. Int. J. Biol. Macromol..

[3] Liang J., Li H., Chen J., He L., Du X., Zhou L., Xiong Q., Lai X., Yang Y., Huang S. (2019). *Dendrobium officinale* polysaccharides alleviate colon tumorigenesis via restoring intestinal barrier function and enhancing anti-tumor immune response. Pharmacol. Res..

[4] Li X., Ding X., Chu B., Zhou Q., Ding G., Gu S. (2008). Genetic diversity analysis and conservation of the endangered Chinese endemic herb *Dendrobium Officinale* Kimura et Migo (Orchidaceae) based on AFLP. Genetica.

[5] Zhang J., Zheng X. (2004). Identification of pathogenic bacteria and cytological study on the infection process of *Dendrobium officinale* black spot. Acta Phytopathol. Sin..

[6] Xiao R., Guan C.-L., Kong D.-D., Li O., Hu X.-F. (2018). Black spot on the medicinal orchid *Dendrobium Officinale* caused by *Cladosporium oxysporum* in China. Can. J. Plant Pathol..

[7] Zhang C., Zhang J., Liu Y., Dai D. (2018). First report of black spot in *Dendrobium officinale* caused by *A. alternata* in Zhejiang Province, China. Plant Dis..

[8] Zhao L. (2020). Pathogen identification and control measures of three diseases of *Dendrobium officinale*. Master’s Thesis.

[9] Wang L., Fang L., Xie J., Wang H. (2017). Preliminary study on etiology of gray mold of *Dendrobium officinale*. Zhejiang J. Agric. Sci..

[10] Xie Y., Wang L., Fang L., Wang H. (2018). First report of leaf spot caused by *Phoma multirostrata* var. *microspora* on *Dendrobium officinale* in Zhejiang Province of China. Plant Dis..

[11] Cao P., Fang Y., Zheng Z., Han X., Zou H., Yan X. (2022). Occurrence of *Neopestalotiopsis clavispora* causing leaf spot on *Dendrobium officinale* in China. Plant Dis..

[12] Sun C., Wang T., Shen X., Wang G., Gao Q., Lou B., Shao Y. (2017). First report of leaf spot caused by *Cladosporium cladosporioides* on *Dendrobium officinale* in China. Plant Dis..

[13] Liu G., Zhao N., Zhao G. (2017). Study on Pathogenic bacteria and pathogenesis of *Dendrobium officinale* dieback disease. J. Southwest For. Univ..

[14] Mirghasempour S.A., Michailides T., Chen W., Mao B. (2022). *Fusarium* Spp. associated with *Dendrobium officinale* dieback disease in China. J. Fungi.

[15] Guo M., Li B., Wang R., Liu P., Chen Q. (2020). Occurrence of dieback disease caused by *Fusarium equiseti* on *Dendrobium officinale* in China. Crop Prot..

[16] Zhao G., Liu G., Zhao N. (2016). Pathogen identification of stem rot from *Dendrobium officinale* of China. Jiangsu J. Agric. Sci..

[17] Cao P., Zheng Z., Fang Y., Han X., Zou H., Yan X. (2022). First report of stem rot caused by *Fusarium kyushuense* on *Dendrobium officinale* in China. Plant Dis..

[18] Li H., Lin J., Wang W., Zou H., Dai Y. (2015). Isolation, identification and screening of bacteriostatic agents of *Dendrobium officinale*. Fujian J. Agric. Sci..

[19] Kong Q., Yuan S., Guo J., Xue C., Li X., Wang Y., Wang T. (2018). Identification and biological characteristics of pathogenic bacteria from *Dendrobium officinale* root rot. Chin. Med..

[20] Zhang Y., Lin B., Zou M., Liang J., Hu H. (2017). First report of *Fusarium* wilt of *Dendrobium officinale* caused by *Fusarium oxysporum* in China. Plant Dis..

[21] Shen W., Zhao L., Wang G., Qiu C., Wang X., Lou B. (2018). Identification of ring rot of *Dendrobium officinale* and screening of fungicides. J. Plant Prot..

[22] Xiao C., Li R., Song X., Tian X., Zhao Q. (2022). First report of soft rot on *Dendrobium officinale* caused by *Epicoccum sorghinum* in China. Plant Dis..

[23] Xiao C., Li R. (2021). Detection and control of *Fusarium oxysporum* from soft rot in *Dendrobium officinale* by loop-mediated isothermal amplification assays. Biology.

[24] Saberi Riseh R., Skorik Y.A., Thakur V.K., Moradi Pour M., Tamanadar E., Noghabi S.S. (2021). Encapsulation of plant biocontrol bacteria with alginate as a main polymer material. Int. J. Mol. Sci..

[25] Ochiai N., Fujimura M., Oshima M., Motoyama T., Ichiishi A., Yamada-Okabe H., Yamaguchi I. (2002). Effects of iprodione and fludioxonil on glycerol synthesis and hyphal development in candida albicans. Biosci. Biotechnol. Biochem..

[26] Nicoletti G., Domalewska E., Borland R. (1999). Fungitoxicity of oxine and copper oxinate: Activity spectrum, development of resistance and synergy. Mycol. Res..

[27] Avenot H.F., Michailides T.J. (2010). Progress in understanding molecular mechanisms and evolution of resistance to succinate dehydrogenase inhibiting (SDHI) fungicides in phytopathogenic fungi. Crop Prot..

[28] Li Y., Ma X., Lu G. (2013). Systematic investigation of the toxic mechanism of difenoconazole on protein by spectroscopic and molecular modeling. Pestic. Biochem. Physiol..

[29] Crous P.W., Lombard L., Sandoval-Denis M., Seifert K., Schroers H.-J., Chaverri P., Gené J., Guarro J., Hirooka Y., Bensch K. (2021). *Fusarium*: More than a node or a foot-shaped basal cell. Stud. Mycol..

[30] Massicotte H., Melville L., Peterson R. (2005). Structural characteristics of root fungal interactions for five ericaceous species in Eastern Canada. Can. J. Bot..

[31] Guo L., Hyde K., Liew E. (1998). A method to promote sporulation in palm endophytic fungi. Fungal Divers..

[32] Pan L., Cui C., Wang B. (2010). A rapid DNA extraction method for filamentous fungi for PCR amplification. J. Microbiol..

[33] Valenzuela-Lopez N., Cano-Lira J., Guarro J., Sutton D.A., Wiederhold N., Crous P., Stchigel A. (2018). Coelomycetous Dothideomycetes with emphasis on the families Cucurbitariaceae and Didymellaceae. Stud. Mycol..

[34] White T.J., Bruns T., Lee S., Taylor J. (1990). Amplification and direct sequencing of fungal ribosomal RNA genes for phylogenetics. PCR Protoc. Guide Methods Appl..

[35] De Hoog G., Van den Ended A. (1998). Molecular diagnostics of clinical strains of filamentous basidiomycetes. Mycoses.

[36] Vilgalys R., Hester M. (1990). Rapid genetic identification and mapping of enzymatically amplified ribosomal DNA from several cryptococcus species. J. Bacteriol..

[37] Rehner S.A., Samuels G.J. (1994). Taxonomy and phylogeny of gliocladium analysed from nuclear large subunit ribosomal DNA sequences. Mycol. Res..

[38] Glass N.L., Donaldson G.C. (1995). Development of primer sets designed for use with the PCR to amplify conserved genes from filamentous ascomycetes. Appl. Environ. Microbiol..

[39] Liu Y.J., Whelen S., Hall B.D. (1999). Phylogenetic relationships among ascomycetes: Evidence from an RNA polymerse II subunit. Mol. Biol. Evol..

[40] Sung G.-H., Sung J.-M., Hywel-Jones N.L., Spatafora J.W. (2007). A multi-gene phylogeny of Clavicipitaceae (Ascomycota, fungi): Identification of localized incongruence using a combinational bootstrap approach. Mol. Phylogenet. Evol..

[41] Chen Q., Jiang J., Zhang G., Cai L., Crous P.W. (2015). Resolving the *Phoma* enigma. Stud. Mycol..

[42] Hou L., Groenewald J., Pfenning L., Yarden O., Crous P.W., Cai L. (2020). The *Phoma*-like dilemma. Stud. Mycol..

[43] Katoh K., Standley D.M. (2013). MAFFT multiple sequence alignment software version 7: Improvements in performance and usability. Mol. Biol. Evol..

[44] Wang Q.-H., Fan K., Li D.-W., Han C.-M., Qu Y.-Y., Qi Y.-K., Wu X.-Q. (2020). Identification, virulence and fungicide sensitivity of *Colletotrichum gloeosporioides* ss responsible for walnut anthracnose disease in China. Plant Dis..

[45] Qi J. (2021). Identification of Pathogenic Bacteria of Dendrobium Officinale Stem Rot and Study on Prevention and Growth of Bacillus velezensis D-12. Master’s Thesis.

[46] Borman A.M., Johnson E.M. (2021). Sequence-based identification and classification of fungi. Trends Syst. Bact. Fungi.

[47] Glaeser S.P., Kämpfer P. (2015). Multilocus sequence analysis (MLSA) in prokaryotic taxonomy. Syst. Appl. Microbiol..

[48] Kashyap A.S., Manzar N., Ahamad F., Tilgam J., Sharma P.K., Saxena A.K. (2022). First report of root rot disease in green gram (*Vigna radiata*) caused by *Ectophoma multirostrata* in India. Plant Dis..

[49] Harteveld D., Akinsanmi O., Drenth A. (2013). Multiple *Alternaria* species groups are associated with leaf blotch and fruit spot diseases of apple in Australia. Plant Pathol..

[50] Ali S., Abbasi P., Rehman S., Ellouze W. (2021). First report of moldy core of sweet tango apples from New Zealand caused by *Alternaria arborescens*. Plant Dis..

[51] Serdani M., Kang J.-C., Andersen B., Crous P.W. (2002). Characterisation of *Alternaria* species-groups associated with core rot of apples in South Africa. Mycol. Res..

[52] Ntasiou P., Myresiotis C., Konstantinou S., Papadopoulou-Mourkidou E., Karaoglanidis G.S. (2015). Identification, Characterization and mycotoxigenic ability of *Alternaria* spp. causing core rot of apple fruit in Greece. Int. J. Food Microbiol..

[53] Matić S., Tabone G., Garibaldi A., Gullino M.L. (2020). *Alternaria* leaf spot caused by *Alternaria* species: An emerging problem on ornamental plants in Italy. Plant Dis..

[54] Akhtar N., Bashir U., Mushtaq S. (2014). First report of leaf spot of rice caused by *Alternaria arborescens* in Pakistan. Plant Dis..

[55] Akram W., Li G., Ahmad A., Anjum T., Ali B., Luo W., Guo J., Xie D., Wang Q. (2019). Leaf spot disease caused by *Alternaria arborescens*, *A. tenuissima*, and *A. infectoria* on *Brassica rapa* subsp. *parachinensis* in China. Plant Dis..

[56] Garibaldi A., Bertetti D., Matić S., Luongo I., Gullino M. (2020). First report of leaf spots caused by *Alternaria arborescens* on *symphyotrichum novi-belgii* in Italy. Plant Dis..

[57] Razak N.J., Abass M.H. (2021). First report of *Alternaria arborescens* causing early blight on tomato in Iraq: Short notes. Basrah J. Agric. Sci..

[58] Aloi F., Riolo M., Sanzani S.M., Mincuzzi A., Ippolito A., Siciliano I., Pane A., Gullino M.L., Cacciola S.O. (2021). Characterization of *Alternaria* species associated with heart rot of pomegranate fruit. J. Fungi.

[59] Luo Y., Hou L., Förster H., Pryor B., Adaskaveg J. (2017). Identification of *Alternaria* species causing heart rot of pomegranates in California. Plant Dis..

[60] Huang X., Luo J., Li B., Song Y., Mu W., Liu F. (2019). Bioactivity, physiological characteristics and efficacy of the SDHI fungicide pydiflumetofen against *Sclerotinia sclerotiorum*. Pestic. Biochem. Physiol..

[61] Chen J., Hu H., Lai R., Wu R. (2018). Field control effect of 18% flumazoylhydroxylamine phenoxymethyclozole suspension on citrus scab. Pesticide.

[62] Zhou Y., Han X., Guo Z., Wang S., Li J., Zhang S. (2021). Evaluation of field control effect of 200g/L fluzoylhydroxylamine and phenoxymethyclozole suspension on cucumber powdery mildew. J. Chang. Veg..

