# Supplementary material for: Characterization and Control of Dendrobium officinale Bud Blight Disease
Source: Pathogens. 2023 Apr 20;12(4):621. doi: 10.3390/pathogens12040621 (PMC10142839; doi:10.3390/pathogens12040621)
Supplement: Supplementary file 1 [file pathogens-12-00621-s001.zip › Table S1-3_Species' accession numbers.pdf]

Table S1. GenBank accession numbers of strains used to identify *Ectophoma* strain in the phylogenetic analyses.

Species	Strain number	GenBank accession numbers			
		ITS	LSU	*tub2*	*rpb2*
Allocucurbitaria botulispora CBS 142452	CBS 142452	LT592932	LN907416	LT593001	LT593070
Allophoma cylindrispora CBS 142453	CBS 142453	LT592920	LN907376	LT592989	LT593058
A. minor CBS 325.82	CBS 325.82	GU237831	GU238107	GU237632	KT389553
A. nicaraguensis CBS 506.91	CBS 506.91	GU237876	GU238058	GU237596	KT389551
A. oligotrophica CBS 497.91	CBS 497.91	KY742040	KY742194	KY742282	KY742128
A. piperis CBS 268.93	CBS 268.93	GU237816	GU238129	GU237644	KT389554
A. tropica CBS 436.7	CBS 436.7	GU237864	GU238149	GU237663	KT389556
Alternariaster bidentis CBS 134021	CBS 134021	KC609333	KC609341	/	KC609347
Briansuttonomyces eucalypti CBS 114879	CBS 114879	KU728479	KU728519	KU728595	/
Calophoma clematidina CBS 108.79	CBS 108.79	FJ426989	FJ515632	FJ427100	KT389588
C. clematidis-rectae CBS 507.63	CBS 507.63	FJ515606	FJ515647	FJ515624	KT389589
C. rosae CGMCC 3.18347	CGMCC 3.18347	KY742049	KY742203	KY742291	KY742135
Camarosporidiella aborescentis MFLUCC 14-0604	MFLUCC 14-0604	KP711377	KP711378	/	/
Camarosporidiella aborescentis MFLUCC 14-0238	MFLUCC 14-0238	KP120926	KP120927	/	/
C. aureum MFLUCC 14-0620	MFLUCC 14-0620	KP744436	KP744478	/	/
C. clematidis MFLUCC 13-0336	MFLUCC 13-0336	KJ562213	KJ562188	/	/
Camarosporium quaternatum CBS 142616	CBS 142616	KY929171	KY929136	/	/
Camarosporomyces flavigenus CBS 314.80	CBS 314.80	KY929138	GU238076	/	/
Cucurbitaria berberidis CBS 130007	CBS 130007	LT717673	KC506793	LT717676	LT854936
Cumuliphoma indica CBS 654.77	CBS 654.77	FJ427043	GU238122	FJ427153	LT623261
C. omnivirens CBS 341.86	CBS 341.86	FJ427042	LT623214	FJ427152	LT623260
C. pneumoniae CBS 142454	CBS 142454	LT592925	LN907392	LT592994	/
Didymella aeria CGMCC 3.18353	CGMCC 3.18353	KY742051	KY742205	KY742137	KY742293
D. aquatica CGMCC 3.18349	CGMCC 3.18349	KY742055	KY742209	KY742297	KY742140
D. arachidicola CBS 333.75	CBS 333.75	GU237833	GU237996	GU237554	KT389598
D. aurea CBS 269.93	CBS 269.93	GU237818	GU237999	GU237557	KT389599
D. boeremae CBS 109942	CBS 109942	FJ426982	GU238048	FJ427097	KT389600
D. brunneospora CBS 115.58	CBS 115.58	KT389505	KT389723	KT389802	KT389625
D. chloroguttulata CGMCC 3.18351	CGMCC 3.18351	KY742057	KY742211	KY742299	KY742142
D. coffeae-arabicae CBS 123380	CBS 123380	FJ426993	GU238005	FJ427104	KT389603
D. ellipsoidea CGMCC 3.18350	CGMCC 3.18350	KY742060	KY742214	KY742302	KY742145
D. exigua CBS 183.55	CBS 183.55	GU237794	EU754155	GU237525	EU874850
D. gardeniae CBS 626.68	CBS 626.68	FJ427003	GQ387595	FJ427114	KT389606
D. glomerata CBS 528.66	CBS 528.66	FJ427013	JX681105	FJ427124	GU371781

D. heteroderae CBS 109.92	CBS 109.92	FJ426983	GU238002	FJ427098	KT389601
D. ilicicola CGMCC 3.18355	CGMCC 3.18355	KY742065	KY742219	KY742307	KY742150
D. infuscatispora CGMCC 3.18356	CGMCC 3.18356	KY742067	KY742221	KY742309	/
D. keratinophila CBS 143032	CBS 143032	LT592901	LN907343	LT592970	LT593039
D. macrophylla CGMCC 3.18357	CGMCC 3.18357	KY742070	KY742224	KY742312	KY742154
D. maydis CBS 588.69	CBS 588.69	FJ427086	EU754192	FJ427190	GU371782
D. microchlamydospora CBS 105.95	CBS 105.95	FJ427028	GU238104	FJ427138	KP330424
D. ocimicola CGMCC 3.18358	CGMCC 3.18358	KY742078	KY742232	KY742320	/
D. pedeiae CBS 124517	CBS 124517	GU237770	GU238127	GU237642	/
D. pinodes CBS 525.77	CBS 525.77	GU237883	GU238023	GU237572	KT389614
D. pomorum CBS 285.76	CBS 285.76	FJ427053	GU238025	FJ427163	KT389615
D. protuberans CBS 381.96	CBS 381.96	GU237853	GU238029	GU237574	KT389620
D. rumicicola CBS 683.79	CBS 683.79	KT389503	KT389721	KT389800	KT389622
D. sancta CBS 281.83	CBS 281.83	FJ427063	GU238030	FJ427170	KT389623
D. segeticola CGMCC 3.17489	CGMCC 3.17489	KP330443	KP330455	KP330399	KP330414
D. suiyangensis CGMCC 3.18352	CGMCC 3.18352	KY742089	KY742243	KY742330	KY742168
Dothidotthia symphoricarpi CBS 119687	CBS 119687	/	EU673273	/	/
Ectophoma multirostrata CBS 274.60	CBS 274.60	FJ427031	GU238111	FJ427141	LT623265
E. pomi CBS 267.92	CBS 267.92	GU237814	GU238128	GU237643	LT623263
Epicoccum brasiliense CBS 120105	CBS 120105	GU237760	GU238049	GU237588	KT389627
Epicoccum camelliae CGMCC 3.18343	CGMCC 3.18343	GU237760	GU238049	GU237588	KY742170
E. catenisporum CBS 181.80	CBS 181.80	FJ427069	LT623213	FJ427175	LT623253
E. dendrobii CGMCC 3.18359	CGMCC 3.18359	KY742093	KY742247	KY742335	/
E. duchesneae CGMCC 3.18345	CGMCC 3.18345	KY742095	KY742249	KY742337	/
E. hordei	CGMCC 3.18360	KY742097	KY742251	KY742339	
E. huancayense	CBS 105.80	GU237732	GU238084	GU237615	*KT389630*
E. italicum	CGMCC 3.18361	KY742099	KY742253	KY742341	*KY742172*
E. keratinophilum	CBS 142455	LT592930	LN907414	LT592999	*LT593068*
E. latusicollum	CGMCC 3.18346	KY742101	KY742255	KY742343	KY742174
E. layuense	CGMCC 3.18362	KY742107	KY742261	KY742349	/
E. nigrum	CBS 173.73	FJ426996	GU237975	FJ427107	KT389632
E. ovisporum	CBS 180.80	FJ427068	LT623212	FJ427174	LT623252
E. plurivorum	CBS 558.81	GU237888	GU238132	GU237647	KT389634
E. pneumoniae	UTHSC:DI16-257; FMR 13747	LT592927	LN907400	LT592996	LT593065
E. poae	CGMCC 3.18363	KY742113	KY742267	KY742355	KY742182
E. proteae	CBS 114179	JQ044433	JQ044452	LT623230	LT623251
E. viticis	CGMCC 3.18344	KY742118	KY742272	KY742360	KY742186
Heterophoma verbascicola	CGMCC 3.18364	KY742119	KY742273	KY742361	KY742187
Leptosphaeria doliolum	CBS 505.75	JF740205	GQ387576	JF740144	KT389640
Libertasomyces myopor	CBS 141302	NR_145200	KX228332	/	/
L. platani	CBS 142112	KY173416	KY173507	KY173604	KY173585

Libertasomyces quercus	CBS 134.97	/	DQ377883	/	/
Macroventuria anomochaeta	CBS 525.71	GU237881	GU237984	GU237544	GU456346
M. wentii	CBS 526.71	GU237884	GU237986	GU237546	KT389642
Neoascochyta argentina	CBS 112524	KT389524	KT389742	KT389822	
N. cylindrispora	CBS 142456	LT592963	LN907502	LT593032	LT593102
N. desmazieri	CBS 297.69	KT389508	KT389726	KT389807	kt389644
N. europaea	CBS 820.84	KT389511	KT389729	KT389809	KT389646
N. paspali	CBS 560.81	FJ427048	GU238124	FJ427158	KP330426
N. soli	CGMCC 3.18365	KY742121	KY742275	KY742363	/
N. tardicrescens	CBS 689.97	KT389526	KT389744	KT389824	KT389654
N. triticicola	CBS 544.74	GU237887	EU754134	GU237488	KT389652
Neocamarosporium goegapense	CBS 138008	KJ869163	KJ869220	/	/
Neocucurbitaria aquatica	CBS 297.74	LT623221	EU754177	LT623238	LT623278
N. cava	CBS 257.68	JF740260	EU754199	KT389844	LT717681
N. hakeae	CBS 142109	KY173436	KY173526	KY173613	KY173593
N. irregularis	CBS 142791	LT592916	LN907372	LT592985	LT593054
N. keratinophila	CBS 121759	EU885415	LT623215	LT623236	LT623275
N. quercina	CBS 115095	LT623220	GQ387619	LT623237	LT623277
Neodidymelliopsis achlydis	CBS 256 77	KT389531	KT389749	KT389829	/
N. longicolla	CBS 382 96	KT389532	KT389750	KT389830	
N. polemonii	CBS 109181	GU237746	GU238133	KT389828	KP330427
N. xanthina	CBS 383.68	GU237855	GU238157	KT389831	KP330431
Neomicrosphaeriopsis italica	MFLUCC 15-0485	KU900318	KU729854	/	KU674820
Neophaeosphaeria agaves	CBS 136429	NR_137833	KF777227	/	/
Neoplatysporoides aloicola	CBS 139901	KR476719	KR476754	/	/
Neopyrenochaeta acicola	CBS 812.95	LT623218	GQ387602	LT623232	LT623271
N. fragariae	CBS 101634	LT623217	GQ387603	LT623231	LT623270
N. inflorescentiae	CBS 119222	EU552153	EU552153	LT623233	LT623272
N. telephoni	CBS 139022	KM516291	KM516290	LT717678	LT717685
Neopyrenochaetopsis hominis	CBS 143033	LT592923	LN907381	LT592992	LT593061
Nothophoma anigozanthi	CBS 381.91	GU237852	GU238039	GU237580	KT389655
N. infossa	CBS 123395	FJ427025	GU238089	FJ427135	KT389659
N. macrospora	CBS 140674	LN880536	LN880537	LN880539	LT593073
N. variabilis	CBS 142457	LT592939	LN907428	LT593008	LT593078
O. frigidarii	CBS 103.81	EU040234	EU040234	/	/
P. camelliae	CGMCC 3.18106	KX829034	KX829042	KX829058	KX829050
P. litseae	CGMCC 3.18109	KX829029	KX829037	KX829053	KX829045
P. oligotrophica	CGMCC 3.18111	KX829031	KX829039	KX829055	KX829047
P. selaginellae	CBS 122.93	GU237762	GU238142	GU237656	LT623255
Paraconiothyrium estuarinum	CBS 109850	JX496016	JX496129	JX496355	LT854937
Paracucurbitaria italica	CBS 234.92	LT623219	EU754176	LT623235	LT623274
Parapyrenochaeta acaciae	CBS 141291	KX228265	KX228316	LT717679	LT717686
P. protearum	CBS 131315	JQ044434	JQ044453	LT717677	LT717683

Phaeosphaeria oryzae	CBS 110110	KF251186	KF251689	KF252680	/
Phomatodes aubrietiae	CBS 627.97	GU237895	GU238045	GU237585	KT389665
Pleospora herbarum	CBS 191.86	NR_111243	JX681120	/	KC584471
Pseudoascochyta novae-zelandiea	CBS 141689	LT592892	LT592893	LT592894	LT592895
P. pratensis	CBS 141688	LT223130	LT223131	LT223132	LT223133
Pseudopyrenochaeta lycopersici	CBS 306.65	NR_103581	EU754205	LT717674	LT717680
P. terretris	CBS 282.72	LT623228	LT623216	LT623246	LT623287
Pyrenochaeta nobilis	CBS 407.76	EU930011	EU754206	KT389845	LT623276
P. botulispora	CBS 142458	LT592946	LN907441	LT593015	LT593085
P. confluens	CBS 142459	LT592950	LN907446	LT593019	LT593089
P. decipiens	CBS 343.85	LT623223	GQ387624	LT623240	LT623280
P. globosa	CBS 143034	LT592934	LN907418	LT593003	LT593072
P. indica	CBS 124454	LT623224	GQ387626	LT623241	LT623281
P. leptospora	CBS 101635	JF740262	GQ387627	LT623242	LT623282
P. microspora	CBS 102876	LT623226	GQ387631	LT623244	LT623284
P. paucisetosa	CBS 142460	LT592897	LN907336	LT592966	LT593035
P. poae	CBS 136769	KJ869117	KJ869175	KJ869243	LT623286
P. setosissima	CBS 119739	LT623227	GQ387632	LT623245	LT623285
P. tabarestanensis	CBS 139506	KF730241	KF803343	KX789523	/
P. uberiformis	CBS 142461	LT592935	LN907420	LT593004	LT593074
Remotididymella anthropophila	CBS 142462	LT592936	LN907421	LT593005	LT593075
R. destructiva	CBS 378.73	GU237849	GU238063	GU237601	LT623258
Similiphoma crystallifera	CBS 193.82	GU237797	GU238060	GU237598	LT623267
Stagonosporopsis dorenboschii	CBS 426.90	GU237862	GU238185	GU237690	KT389678
Staurosphaeria aloes	CBS 136437	NR_137821	KF777198	/	/
S. aptrootii	CBS 483.95	KY929149	GU301806	/	/
S. lyciicola	CBS 142619	KY929150	KY929180	/	/
Vacuiphoma bulgarica	CBS 357.84	GU237837	GU238050	GU237589	LT623256
V. oculihominis	UTHSC:DI16-308; FMR13801	LT592954	LN907451	LT593023	LT593093
X. asphodeli	CBS 375.62	KT389549	KT389765	KT389853	KT389689
X. saxea	CBS 419.92	GU237860	GU238141	GU237655	KP330429
Xenopyrenochaetopsis pratorum	CBS 445.81	JF740263	GU238136	KT389846	KT389671

CBS: Westerdijk Fungal Biodiversity Institute (WIFB), Utrecht, The Netherlands. NRRL (Northern Regional Research Laboratory): Agricultural Research Service Culture Collection Database, Peoria, USA. CMW: The working collection of FABI (Forestry and Agricultural Biotechnology Institute), University of Pretoria, South Africa. BBA: Julius Kühn-Institute, Institute for Epidemiology and Pathogen Diagnostics, Berlin and Braunschweig, Germany. CPC: Collection of P.W. Crous.

Table S2. GenBank accession numbers used to identify *Alternaria* strains used in the phylogenetic analyses.

Species	Strain number	GenBank accession number		
		ITS	LSU	*rpb2*
Alternaria abundans	CBS 534.83	JN383485	KC584323	KC584448
A. A.e	CBS 126989	AF229485	KC584346	KC584470

A. alternata	CBS 916.96	AF347031	DQ678082	KC584375
A. anigozanthi	CBS 121920	KC584180	KC584252	KC584376
A. arborescens	CBS 102605	AF347033	KC584253	KC584377
A. argyranthemi	CBS 116530	KC584181	KC584254	KC584378
A. armoraciae	CBS 118702	KC584182	KC584255	KC584379
A. aspera	CBS 115269	KC584242	KC584349	KC584474
A. atra	CBS 195.67	AF229486	KC584350	KC584475
A. avenicola	CBS 121459	KC584183	KC584256	KC584380
A. axiaeriisporifera	CBS 118715	KC584184	KC584257	KC584381
A. bornmuelleri	DAOM 231361	FJ357317	KC584366	KC584491
A. botryospora	CBS 478.90	AY278844	KC584336	KC584461
A. botrytis	CBS 197.67	KC584243	KC584351	KC584476
A. brassicae	CBS 116528	KC584185	KC584258	KC584382
A. brassicae- pekinensis	CBS 121493	KC584244	KC584353	KC584478
A. brassicicola	CBS 118699	JX499031	KC584259	KC584383
A. breviramosa	CBS 121331	FJ839608	KC584318	KC584442
A. caespitosa	CBS 177.80	KC584250	KC584367	KC584492
A. calycipyricola	CBS 121545	KC584186	KC584260	KC584384
A. cantlous	CBS 123007	KC584245	KC584354	KC584479
A. caricis	CBS 480.90	AY278839	KC584342	KC584467
A. carotiincultae	CBS 109381	KC584188	KC584262	KC584386
A. cetera	CBS 121340	JN383482	KC584317	KC584441
A. chartarum	CBS 200.67	AF229488	KC584356	KC584481
A. cheiranthi	CBS 109384	AF229457	KC584263	KC584387
A. chlamydospora	CBS 491.72	KC584189	KC584264	KC584388
A. chlamydosporigena	CBS 341.71	KC584231	KC584326	KC584451
A. cinerariae	CBS 116495	KC584190	KC584265	KC584389
A. concatenata	CBS 120006	KC584246	KC584355	KC584480
A. conjuncta	CBS 196.86	FJ266475	KC584266	KC584390
A. consortialis	CBS 104.31	KC584247	KC584357	KC584482
A. cucurbitae	CBS 483.81	FJ266483	KC584358	KC584483
A. cumini	CBS 121329	KC584191	KC584267	KC584391
A. dauci	CBS 117097	KC584192	KC584268	KC584392
A. daucifolii	CBS 118812	KC584193	KC584269	KC584393
A. dennisii	CBS 476.90	JN383488	KC584329	KC584454
A. dianthicola	CBS 116491	KC584194	KC584270	KC584394
A. elegans	CBS 109159	KC584195	KC584271	KC584395
A. ellipsoidea	CBS 119674	KC584196	KC584272	KC584396
A. embellisia	CBS 339.71	KC584230	KC584324	KC584449
A. eryngii	CBS 121339	JQ693661	KC584273	KC584397
A. ethzedia	CBS 197.86	AF392987	KC584274	KC584398
A. eureka	CBS 193.86	JN383490	KC584331	KC584456
A. gaisen	CBS 632.93	KC584197	KC584275	KC584399

A. geniostomatis	CBS 118701	KC584198	KC584276	KC584400
A. gypsophilae	CBS 107.41	KC584199	KC584277	KC584401
A. helianthiinficiens	CBS 117370	KC584200	KC584278	KC584402
A. helianthiinficiens	CBS 208.86	JX101649	KC584279	KC584403
A. heterospora	CBS 123376	KC584248	KC584363	KC584488
A. hyacinthi	CBS 416.71	KC584233	KC584332	KC584457
A. indefessa	CBS 536.83	KC584234	KC584333	KC584458
A. infectoria	CBS 210.86	DQ323697	KC584280	KC584404
A. japonica	CBS 118390	KC584201	KC584281	KC584405
A. juxtiseptata	CBS 119673	KC584202	KC584282	KC584406
A. leptinellae	CBS 477.90	KC584235	KC584334	KC584459
A. leucanthemi	CBS 422.65	KC584241	KC584348	KC584473
A. leucanthemi	CBS 421.65	KC584240	KC584347	KC584472
A. limaciformis	CBS 481.81	KC584203	KC584283	KC584407
A. limoniasperae	CBS 102595	FJ266476	KC584284	KC584408
A. lolii	CBS 115266	JN383492	KC584335	KC584460
A. longipes	CBS 540.94	AY278835	KC584285	KC584409
A. macrospora	CBS 117228	KC584204	KC584286	KC584410
A. mimicula	CBS 118696	FJ266477	KC584287	KC584411
A. molesta	CBS 548.81	KC584205	KC584288	KC584412
A. mouchaccae	CBS 119671	KC584206	KC584289	KC584413
A. multiformis	CBS 102060	FJ266486	KC584359	KC584484
A. nepalensis	CBS 118700	KC584207	KC584290	KC584414
A. nobilis	CBS 116490	KC584208	KC584291	KC584415
A. obclavata	CBS 124120	KC584225	FJ839651	KC584443
A. oregonensis	CBS 542.94	FJ266478	KC584292	KC584416
A. oudemansii	CBS 114.07	FJ266488	KC584361	KC584486
A. panax	CBS 482.81	KC584209	KC584293	KC584417
A. papavericola	CBS 116606	FJ357310	KC584321	KC584446
A. penicillata	CBS 116608	FJ357311	KC584316	KC584440
A. penicillata	CBS 116607	KC584229	KC584322	KC584447
A. perpunctulata	CBS 115267	KC584210	KC584294	KC584418
A. petroselini	CBS 112.41	KC584211	KC584295	KC584419
A. photistica	CBS 212.86	KC584212	KC584296	KC584420
A. phragmospora	CBS 274.70	JN383493	KC584337	KC584462
A. planifunda	CBS 537.83	FJ357315	KC584338	KC584463
A. porri	CBS 116698	DQ323700	KC584297	KC584421
A. proteae	CBS 475.90	AY278842	KC584339	KC584464
A. pseudorostrata	CBS 119411	JN383483	KC584298	KC584422
A. radicina	CBS 245.67	KC584213	KC584299	KC584423
A. saponariae	CBS 116492	KC584215	KC584301	KC584425
A. scirpicola	CBS 481.90	KC584237	KC584344	KC584469
A. selini	CBS 109382	AF229455	KC584302	KC584426

A. septorioides	CBS 106.41	KC584216	KC584303	KC584427
A. simsimi	CBS 115265	JF780937	KC584304	KC584428
A. slovaca	CBS 567.66	KC584226	KC584319	KC584444
A. smyrnii	CBS 109380	AF229456	KC584305	KC584429
A. solani	CBS 116651	KC584217	KC584306	KC584430
A. soliaridae	CBS 118387	KC584218	KC584307	KC584431
A. solidaccana	CBS 118698	KC584219	KC584308	KC584432
A. sonchi	CBS 119675	KC584220	KC584309	KC584433
A. subcucurbitae	CBS 121491	KC584249	KC584364	KC584489
A. tagetica	CBS 479.81	KC584221	KC584310	KC584434
A. tellustris	CBS 538.83	FJ357316	KC584340	KC584465
A. tenuissima	CBS 918.96	AF347032	KC584311	KC584435
A. terricola	CBS 202.67	FJ266490	KC584365	KC584490
A. thalictrigena	CBS 121712	EU040211	KC584312	KC584436
A. triglochinicola	CBS 119676	KC584222	KC584313	KC584437
A. tumida	CBS 539.83	FJ266481	KC584341	KC584466
A. vaccariae	CBS 116533	KC584223	KC584314	KC584438
A. vaccariicola	CBS 118714	KC584224	KC584315	KC584439
A.ster helianthi	CBS 119672	/	KC584368	KC584493
Cicatricea salina	CBS 302.84	JN383486	KC584325	KC584450
Paradendryphiella arenariae	CBS 181.58	/	KC793338	DQ470924
Peyronellaea zeae- maydis	CBS 588.69	/	EU754192	GU371782
Pleospora tarda	CBS 714.68	KC584238	KC584345	AF107804
Pyrenochaeta nobilis	CBS 407.76	/	DQ678096	DQ677991
Stemphylium herbarum	CBS 191.86	KC584239	GU238160	KC584471

CBS: Westerdijk Fungal Biodiversity Institute (WIFB), Utrecht, The Netherlands. NRRL (Northern Regional Research Laboratory): Agricultural Research Service Culture Collection Database, Peoria, USA. CMW: The working collection of FABI (Forestry and Agricultural Biotechnology Institute), University of Pretoria, South Africa. BBA: Julius Kühn-Institute, Institute for Epidemiology and Pathogen Diagnostics, Berlin and Braunschweig, Germany. CPC: Collection of P.W. Crous.

Table S3. GenBank accession numbers used to identify *Stagonosporopsis* strains used in the phylogenetic analyses.

Species	Strain number	GenBank accession number			
		ITS	LSU	*tub2*	*rpb2*
Allophoma piperis	CBS 268.93	GU237816	GU238129	GU237644	KT389554
S. actaeae	CBS 105.96	GU237733	GU238165	GU237670	MT018018
Stagonosporopsis actaeae	CBS 106.96	GU237734	GU238166	GU237671	KT389672
S. ailanthicola	MFLUCC 16-1439	KY100872	KY100874	KY100878	KY100876
S. ajacis	CBS 176.93	GU237790	GU238167	GU237672	MT018035
S. ajacis	CBS 177.93	GU237791	GU238168	GU237673	KT389673
S. andigena	CBS 101.80	GU237714	GU238169	GU237674	/
S. artemisiicola	CBS 102636	GU237728	GU238171	GU237676	KT389674
S. astragali	CBS 178.25	GU237792	GU238172	GU237677	MT018030

S. bomiensis	LC 8167	KY742123	KY742277	KY742365	KY742189
S. bomiensis	LC 8168	KY742124	KY742278	KY742366	KY742190
S. caricae	CBS 119735	MN973042	MN973431	MN984054	MN983680
S. centaureae	MFLUCC 16-0787	KX611240	KX611238	/	/
S. chrysanthemi	CBS 137.96	GU237783	GU238191	GU237696	MT018011
S. chrysanthemi	CBS 500.63	GU237871	GU238190	GU237695	MT018012
S. citrulli	FLAS-F-58996	KJ855546	/	KJ855602	/
S. crystalliniformis	CBS 713.85	GU237903	GU238178	GU237683	KT389675
S. crystalliniformis	CBS 771.85	GU237906	GU238179	GU237684	/
S. cucumeris	CBS 386.65	MN973455	MN943657	MT005554	MT018021
S. cucurbitacearum	CBS 109171	GU237922	GU238180	GU237685	MN983682
S. cucurbitacearum	CBS 214.65	MN973454	MN943656	MT005553	MT018020
S. cucurbitacearum	CBS 233.52	MN973456	MN943658	MT005555	MT018024
S. dennisii	CBS 135.96	GU237782	GU238183	GU237688	MT018019
S. dennisii	CBS 631.68	GU237899	GU238182	GU237687	KT389677
S. dorenboschii	CBS 320.90	GU237830	GU238184	GU237689	MT018039
S. dorenboschii	CBS 426.90	GU237862	GU238185	GU237690	KT389678
S. helianthi	CBS 155.90	MN973457	MN943659	MT005556	MT018025
S. helianthi	CBS 200.87	KT389545	KT389761	KT389848	KT389683
S. heliopsidis	CBS 109182	GU237747	GU238186	GU237691	KT389679
S. hortensis	CBS 104.42	GU237730	GU238198	GU237703	KT389680
S. hortensis	CBS 572.85	GU237893	GU238199	GU237704	KT389681
S. inoxydabilis	CBS 425.90	GU237861	GU238188	GU237693	KT389682
S. loticola	CBS 562.81	GU237890	GU238192	GU237697	KT389684
S. loticola	CBS 563.81	MN973465	MN943667	MT005564	MT018040
S. lupini	CBS 101494	GU237724	GU238194	GU237699	KT389685
S. nemophilae	CBS 715.85	MN973460	MN943662	MT005559	MT018031
S. oculo-hominis	CBS 634.92	GU237901	GU238196	GU237701	KT389686
S. papillata	CGMCC 3.18367	KY742125	KY742279	KY742367	KY742191
S. pini	MFLUCC 18-1549	MK347800	MK348019	MK412886	MK434860
S. rhizophilae	XDPOP-RS-9	MN422101	MN422103	MN422099	MN422105
S. rudbeckiae	CBS 109180	GU237745	GU238197	GU237702	MT018015
S. sambucella	CBS 130003	MN973459	MN943661	MT005558	MT018029
S. stuijvenbergii	CBS 144953	MN823449	MN823300	MN824623	MN824475
S. tanaceti	CBS 131484	NR_111724	KP161044	JQ897496	MT018013
S. tanaceti	CBS 131485	MN973452	MN943654	MT005551	MT018014
S. trachelii	CBS 379.91	GU237850	GU238173	GU237678	KT389687
S. trachelii	CBS 384.68	GU237856	GU238174	GU237679	MT018016
S. valerianellae	CBS 273.92	GU237819	GU238200	GU237705	MT018033
S. valerianellae	CBS 329.67	GU237832	GU238201	GU237706	MT018034
S. weymaniae	CBS 144959	MN823453	MN823304	MN824627	MN824479

CBS: Westerdijk Fungal Biodiversity Institute (WIFB), Utrecht, The Netherlands. NRRL (Northern Regional Research Laboratory): Agricultural Research Service Culture Collection Database, Peoria, USA. CMW: The working collection of FABI (Forestry and Agricultural Biotechnology Institute), University of Pretoria, South Africa. BBA: Julius Kühn-Institute, Institute for Epidemiology and Pathogen Diagnostics, Berlin and Braunschweig, Germany. CPC: Collection of P.W. Crous.
