# Supplementary figures and images for: Characterization and Control of Dendrobium officinale Bud Blight Disease
Source: Pathogens. 2023 Apr 20;12(4):621. doi: 10.3390/pathogens12040621 (PMC10142839; doi:10.3390/pathogens12040621)

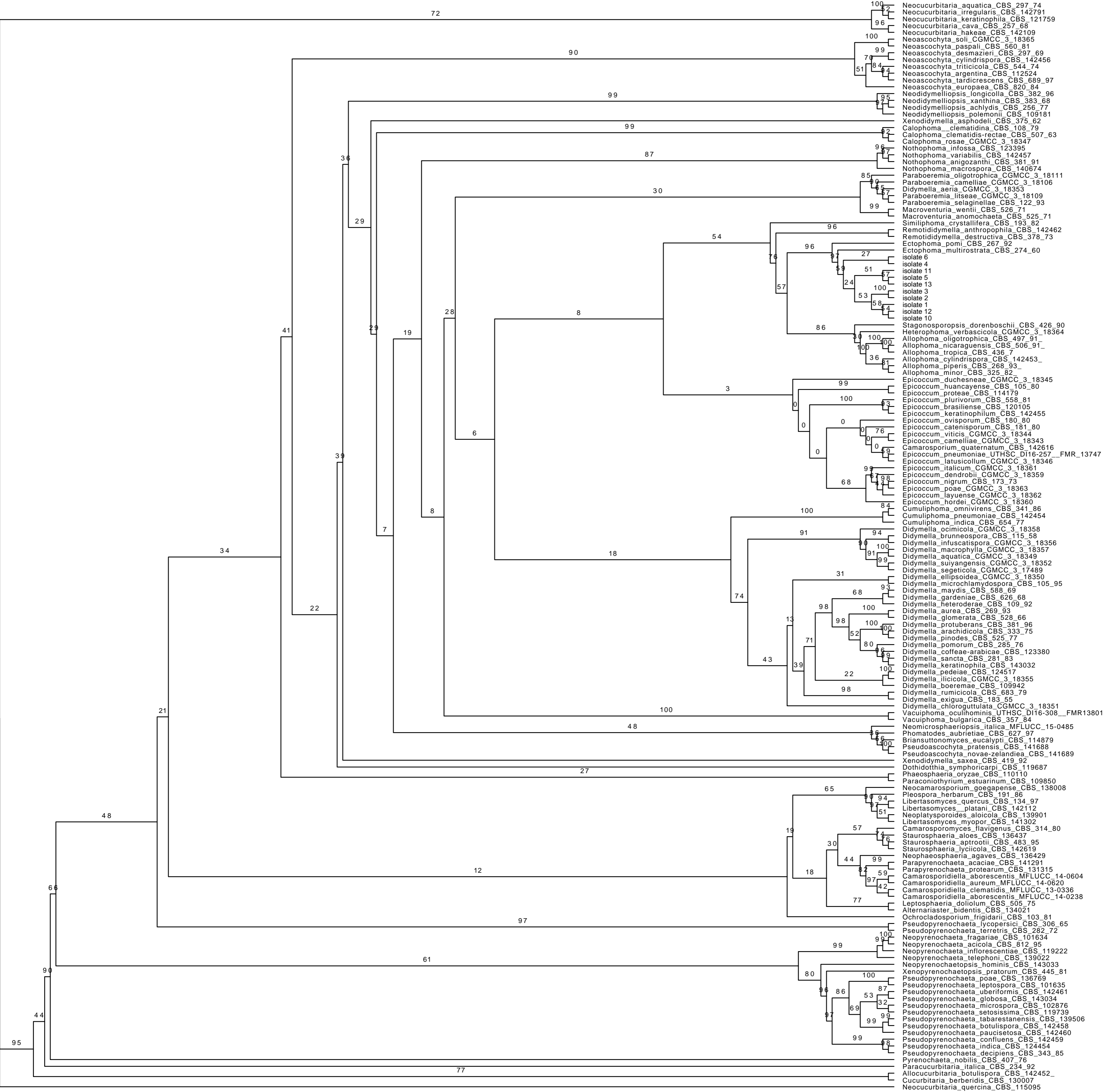

Supplement: Supplementary file 1 [file pathogens-12-00621-s001.zip › Figure S1_PhylogeneticTree for Ectophoma Strains.pdf]

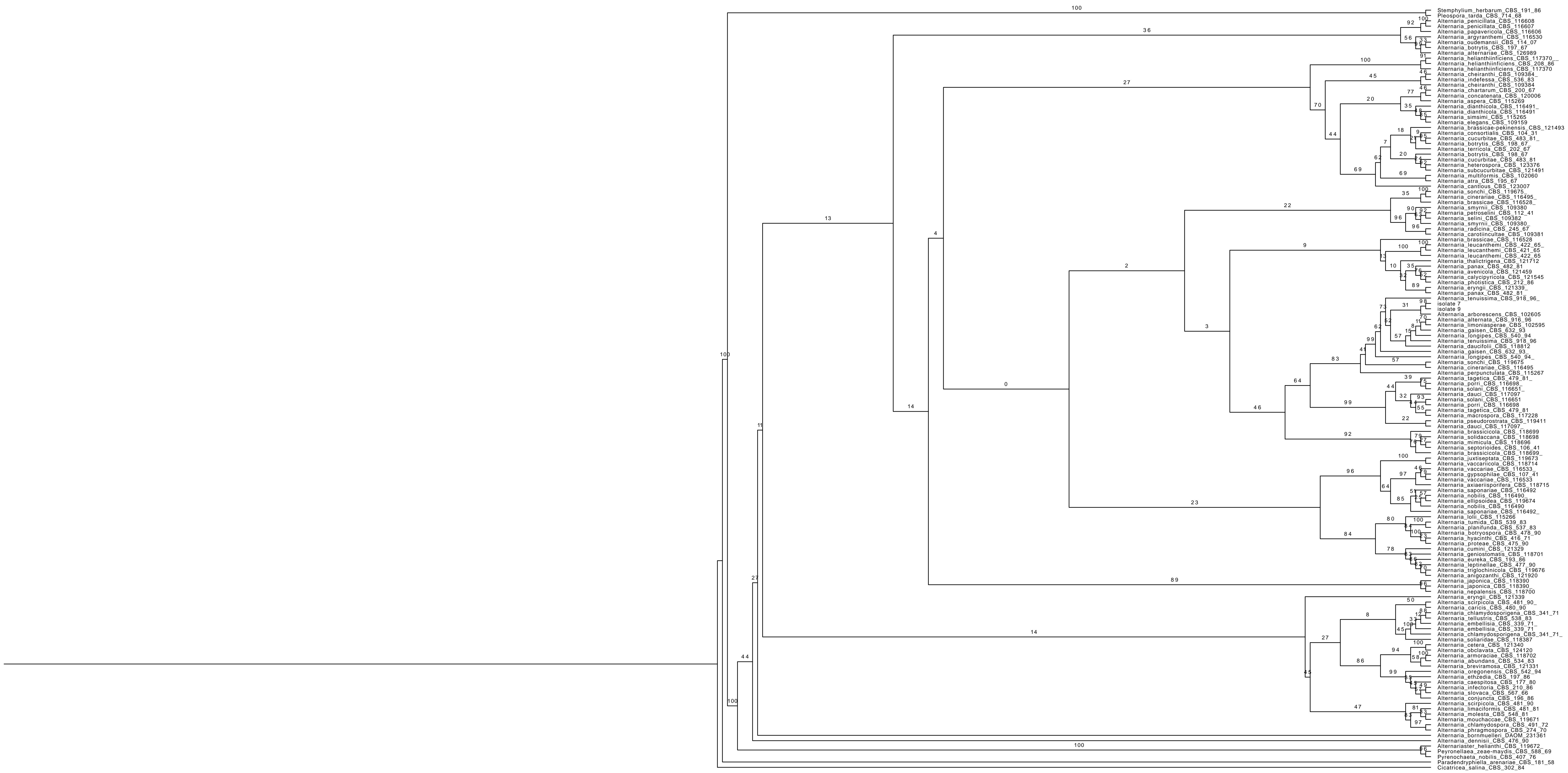

Supplement: Supplementary file 1 [file pathogens-12-00621-s001.zip › Figure S2_PhylogeneticTree for Alternaria Strains.pdf]

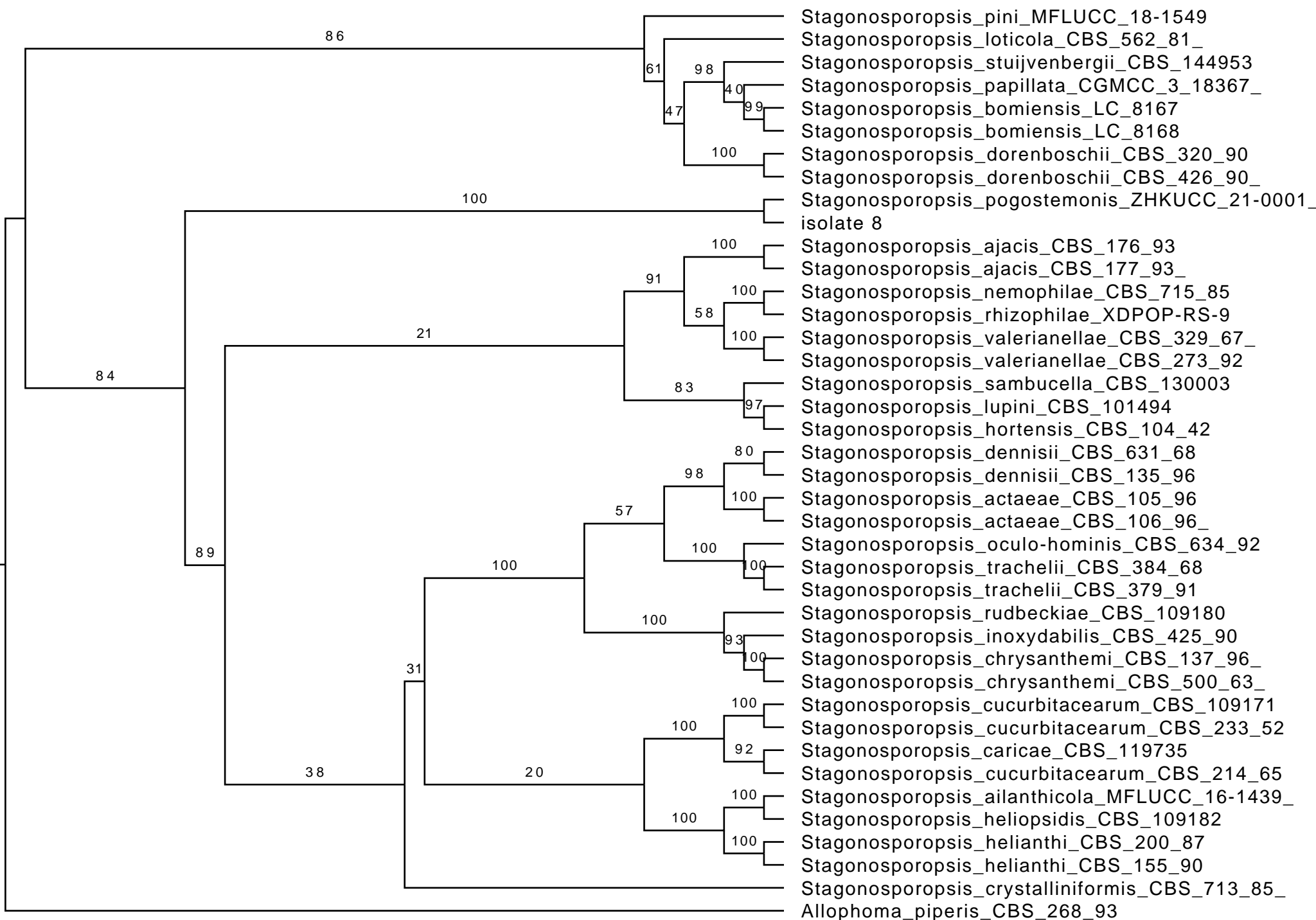

4.0

Supplement: Supplementary file 1 [file pathogens-12-00621-s001.zip › Figure S3_PhylogeneticTree for Stagonosporopsis Strain.pdf]
